# Variability of macrolide-resistant profile in *Mycobacterium avium* complex pulmonary disease

**DOI:** 10.1128/aac.01213-24

**Published:** 2024-10-08

**Authors:** Kiyoharu Fukushima, Yuki Matsumoto, Yuko Abe, Kazuki Hashimoto, Daisuke Motooka, Seigo Kitada, Haruko Saito, Sho Komukai, Eriko Fukui, Takayuki Niitsu, Hiroshi Nabeshima, Yasuharu Nagahama, June Yamauchi, Tadayoshi Nitta, Takuro Nii, Takanori Matsuki, Kazuyuki Tsujino, Keisuke Miki, Yasushi Shintani, Atsushi Kumanogoh, Shizuo Akira, Shota Nakamura, Hiroshi Kida

**Affiliations:** 1 Department of Respiratory Medicine, National Hospital Organization, Osaka Toneyama Medical Center, Toyonaka, Osaka, Japan; 2 Department of Respiratory Medicine and Clinical Immunology, Osaka University Graduate School of Medicine, Suita, Osaka, Japan; 3 Department of Host Defense, Research Institute for Microbial Diseases (RIMD), Osaka University, Suita, Osaka, Japan; 4 Laboratory of Host Defense, World Premier Institute Immunology Frontier Research Center (WPI-IFReC), Osaka University, Suita, Osaka, Japan; 5 Global Center for Medical Engineering and Informatics, Suita, Osaka, Japan; 6 Department of Infection Metagenomics, Genome Information Research Center, Research Institute for Microbial Diseases (RIMD), Osaka University, Suita, Osaka, Japan; 7 Institute for Open and Transdisciplinary Research Initiatives, Osaka University, Osaka, Japan; 8 Kitada Respiratory Clinic, Yao, Osaka, Japan; 9 Department of Clinical Laboratory, National Hospital Organization, Osaka Toneyama Medical Centre, Toyonaka, Osaka, Japan; 10 Department of Biomedical Statistics, Graduate School of Medicine, Osaka University, Osaka, Japan; 11 Department of General Thoracic Surgery, Osaka University Graduate School of Medicine, Suita, Osaka, Japan; 12 Center for Infectious Disease Education and Research, Osaka University, Japan for Infectious Disease Education and Research, Osaka University, Toyonaka, Osaka, Japan; St. George's, University of London, London, United Kingdom

**Keywords:** macrolide resistance, *Mycobacterium avium* complex, non-tuberculous mycobacteria, drug resistance, whole-genome sequence, variable number tandem repeat

## Abstract

This single-center retrospective study aimed to analyze the variability of macrolide resistance (MR) in 68 patients with *Mycobacterium avium* complex pulmonary disease. Among 25 patients treated without macrolides, 13 (52%) reverted to macrolide-susceptible (MS) profiles. Only one (2%) of 43 patients who continued macrolide treatment showed this change. We compared 30 MR isolates with recent specimens. Among them, seven shifted to MS (five attributed to clonally related strains; two resulting from reinfection or polyclonal infection).

## INTRODUCTION

The increasing incidence of *Mycobacterium avium* complex (MAC) pulmonary disease (PD) is a major public health concern (1, 2). Macrolide-containing multidrug combination therapy, a guideline-based therapy, has advanced MAC-PD treatment. However, macrolide resistance (MR) emerges in 9.2–12.0% of patients during guideline-based therapy (3–5). MR emergence is associated with poor outcomes, including mortality (6–8). A point mutation in adenine at position 2058 or 2059 of domain V in the 23S rRNA gene (*rrl*) (6, 9) is responsible for 80–100% of acquired MR cases. As treatment options for MR-MAC-PD are limited, and generally MR is considered an irreversible condition, in clinical practice, the maintenance therapy with macrolides is frequently pursued with the justification that macrolide has favorable immune-modulating effects for bronchiectasis (10–13). However, the benefits of this treatment for MR-MAC-PD have not been clarified (5, 10, 14).

A study comparing mycobacterial strains before and after acquiring MR among patients with AIDS found both scenarios: strains that were clonally different and strains that were clonally identical (15). In the former case, multiple strains with varying susceptibility to macrolide or reinfection of novel strains from environment exist, and macrolide treatment allowed the resistant strains to survive. In the latter case, a clonally related strain, derived from the original strain and with changes in the regions affecting macrolide susceptibility, emerged and was selected within the host. In MR-MAC-PD, it has also been reported that clonality either remained consistent or diverged before and after the acquisition of MR (8, 16). During the course of refractory MAC-PD, relapses with clonally related strains exhibiting a higher minimum inhibitory concentration than the original strain were observed (17), supporting the notion that MAC is adaptable and continues to evolve in response to macrolide pressure.

This single-center retrospective study investigated the long-term variability of MR and the impact of maintenance therapy with macrolides. It included 68 patients newly diagnosed with MR-MAC-PD between January 2012 and June 2022, whose drug susceptibility test (DST) results could be tracked and followed up post-MR emergence. Of these patients, 13 (19.1%) were male, and the median age was 72.0 (interquartile range [IQR], 64.3–78.0). The clarithromycin (CLR) and azithromycin (AZM) groups were combined for analysis as “macrolide treatments” because of their linked drug susceptibilities, whereas erythromycin was excluded because it is not a first-line treatment for MAC-PD and does not induce MR with long-term use (18, 19). Detailed methods are provided in the Supplementary Methods.

**FIG 1:**
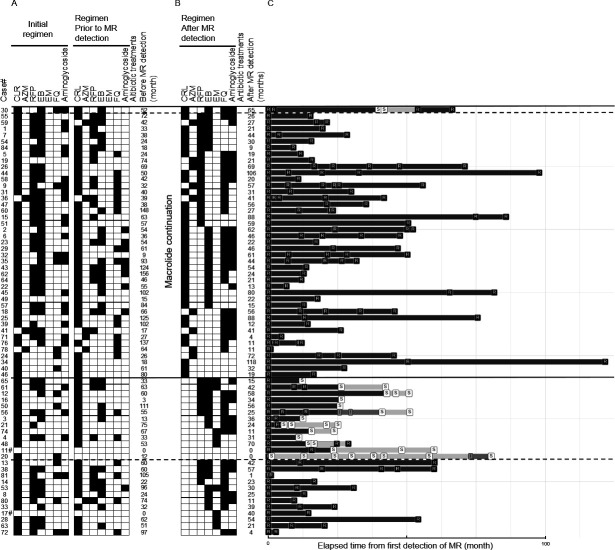
The drug regimen before and after macrolide resistance (MR) profile detection and the time course of the drug susceptibility test (DST) after MR detection. (**A**) Drugs used in the initial treatment and treatment prior to MR detection. Closed cells indicate drugs administered for treatment. Treatment duration from the start of initial treatment to the MR detection is also shown. (**B**) Drugs used in the treatment and treatment duration after MR detection. (**C**) Time course of DST results for CLR from MR detection. Abbreviations: CLR, clarithromycin; EB, ethambutol; EM, erythromycin; FQ, fluoroquinolone; I, intermediate; R, resistant; RFP, rifampicin; S, susceptible. ^#^One patient (#11) had a history of occasional prescription of CLR for symptom aggravation, and another (#17) had a history of multidrug treatment with CLR + levofloxacin.

All 68 patients with MR-MAC-PD had histories of macrolide exposure prior to MR diagnosis (Fig. 1A). Post diagnosis, 43 patients continued macrolide therapy, whereas 25 were treated without macrolides (Fig. 1B). Contrary to our expectations, macrolide susceptibility re-emerged in 13 of the 25 patients treated without macrolides compared with only one of the 43 patients who continued treatment (median, 21.5 months; IQR, 12.0–44.0). Discontinuing macrolide therapy after MR emergence was apparently advantageous for re-emergence of macrolide susceptibilities (Fig. 1C).

Next, we compared 60 MAC isolates from 30 patients. For each patient, the isolate cultured at the time of MR detection was compared with the most recently available isolate. First, we performed a variable number tandem repeat (VNTR) analysis and detected apparent changes in VNTR scores in three patients (10%) (Table 1). Among seven patients who experienced re-emergence of macrolide susceptibility, VNTR changes were observed in patients #11 and #56. This finding suggests that these patients may have been reinfected with a new macrolide-susceptible (MS) strain or experienced predominance of polyclonal strains.

**TABLE 1:** Comparative analysis of MIC, 23SrRNA 2058/2059 mutations, and VNTR in MR-MAC-PD isolates at diagnosis and most recent cultures^*a*^

No.	ML treatment	At MR diagnosis							Most recent						Δ VNTR
		DST		23S rRNA position (2058–2059)					DST		23S rRNA position (2058–2059)				
		MIC		AA	AC	AG	CA	GA		MIC	AA	AC	AG	CA	
2	+	R	>32				100		R	>32	1		1	97	-
5	+	R	>32	100					R	>32				100	+
7	+	R	>32				100		R	>32				100	-
9	+	R	>32			100			R	>32			100		-
15	+	R	>32				100		R	>32				100	-
19	+	R	>32				100		R	>32				100	-
22	+	R	>32		100				R	>32		100			-
24	+	R	>32			100			R	>32			100		-
26	+	R	>32		100				R	>32		100			-
39	+	R	>32	1				99	R	>32		98			-
43	+	R	>32				100		R	>32				100	-
54	+	R	>32			100			R	>32			100		-
55	+	R	>32	100					R	>32	61			39	-
58	+	R	>32		100				R	>32		100			-
60	+	R	>32		100				R	>32	53	47			-
62	+	R	>32		76		24		R	>32		100			-
64	+	R	>32	100					R	>32	100				-
76	+	R	>32		100				R	>32		100			-
84	+	R	>32	45				55	R	>32	43				-
3	-	R	>32		100				S	0.3	99				-
4	-	R	>32	8		92			S	1	100				-
11	-	R	>32	100					S	1	100				+
20	-	R	>32	100					S	8	100				-
48	-	R	>32				100		S	0.5	100				-
56	-	R	>32	100					S	0.3	100				+
65	-	R	>32				100		S	0.1	100				-
8	-	R	>32	100					R	>32	65				-
13	-	R	>32					100	R	>32					-
38	-	R	>32				100		R	>32				100	-
63	-	R	>32			100			R	>32	28		72		-

^*a*^ Abbreviations: ML, macrolide ; DST, drug susceptibility test; MIC, minimum inhibitory concentration of clarithromycin; S, macrolide susceptible; R, macrolide resistant; ΔVNTR, change in variable number of tandem repeats; A, adenine; C, cytosine; G, guanine.

^*b*^ + in ML treatment indicates patients treated with macrolide-containing regimens, - indicates patients treated without macrolide-containing regimens. + in ΔVNTR indicates change in VNTR, - indicates same VNTR.

Subsequently, we examined point mutations in *rrl* gene using whole-genome sequencing. Nine patients (30.0%) exhibited coexistence of wild-type genotypes (AA) and mutant genotypes (AC, AG, CA, GA) either at MR diagnosis or in the most recent isolate (Table 1). In four patients (#3, #4, #48, #65) who experienced re-emergence of macrolide susceptibility, genotypic change at this position occurred without VNTR change. This observation suggests that, in these four patients, a clone closely related to the previously infected clone, indistinguishable by VNTR, has emerged.

**FIG 2:**
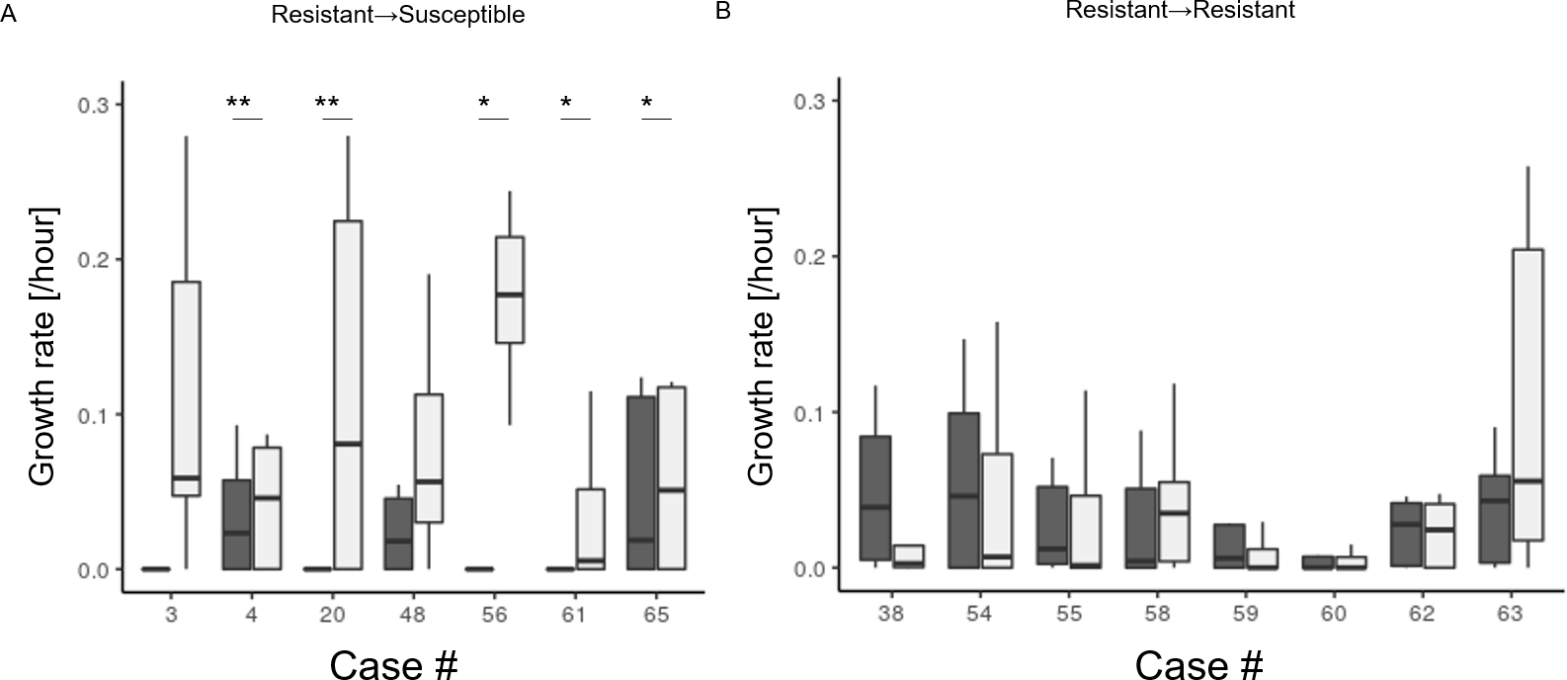
Examination of the growth rate of paired clinical isolates. (**A**) Isolates with reappearance of the macrolide-susceptible profile (*n* = 7). Isolates obtained during macrolide resistance profile (MR) detection and the most recently available isolate are compared among patients who regained macrolide susceptibility. (**B**) Isolates obtained at MR detection and the most recently available isolate are compared among patients who remained MR (*n* = 8). CLR, clarithromycin.

Finally, we compared the *in vitro* proliferative capacities of paired isolates from the same patient. Isolate obtained from each patient from the time of MR detection was compared with the most recent isolate. In patients who regained macrolide susceptibility, the MS isolates exhibited higher *in vitro* growth rates compared with those of the MR isolate (Fig. 2A). Conversely, no differences were observed in patients who had never regained macrolide susceptibility (Fig. 2B). These results suggest that in patients who regained macrolide susceptibility, the MS clones—whether pre-existing alongside the MR strain and surviving through mechanisms such as biofilm formation (20), emerging from the MR strain, or newly acquired from the environment—benefited from the withdrawal of macrolide pressure.

Our findings demonstrate that MR in MAC-PD is reversible, and continuing macrolide impeded the reversion to an MS. The continuation of macrolide in patients with MR needs to be clinically re-evaluated. Additionally, our study revealed that clonally related wild-type and mutant *rrl* genotypes coexist at varying frequencies within MR isolates, suggesting a susceptibility of the *rrl* gene locus to mutations. Upon removal of macrolide pressure, the MS clones might become dominant, leading to a reversion from MR to MS.

This study had certain limitations. First, it was conducted at a single referral center in Japan. Our methods and sample size were insufficient to reach a definitive conclusion, warranting further investigation through larger studies. Second, a delay exists between changes in MAC susceptibility and their detection, as routine re-examinations occur at least once annually after initial MR detection or upon suspicion of recurrence. To minimize the time lag, DST was performed for patients whose stored culture specimens were available. Third, the growth rate of *in vitro* cultures may not correlate with growth *in vivo*; various interactions might occur within the host. Fourth, the evaluation of clonality of bacterial population using VNTR analyses focused on dominant infection strains. VNTR analysis is a well-established and reliable method for differentiation (21–23). We performed technical replicates at least three times, and the results were carefully checked and reviewed by different individuals.

## Data Availability

Whole-genome sequence data were deposited to BioProject (PRJDB13569) and NCBI/ENA/DDBJ (DRX361480 to DRX361528 and DRX484175 to DRX484203).

## SUPPLEMENTAL MATERIAL

Supplemental methods aac.01213-24-s0001.docx Additional experimental details.

ASM does not own the copyrights to Supplemental Material that may be linked to, or accessed through, an article. The authors have granted ASM a non-exclusive, world-wide license to publish the Supplemental Material files. Please contact the corresponding author directly for reuse.
