# Supplementary material for: Variability of macrolide-resistant profile in Mycobacterium avium complex pulmonary disease
Source: Antimicrob Agents Chemother. 2024 Oct 8;68(11):e01213-24. doi: 10.1128/aac.01213-24 (PMC11539230; doi:10.1128/aac.01213-24)
Supplement: Supplemental methods — Additional experimental details. [file aac.01213-24-s0001.docx]

**Supplementary Methods**

***Patients***

The experimental protocol for data involving human participants followed the Ethical Guidelines of the Japan Ministries of Health and Labour for Medical and Health Research Involving Human Subjects. All experiments were conducted in accordance with the principles laid out in the Declaration of Helsinki. The study was approved by the institutional research ethics boards of Osaka Toneyama Medical Center (approval number: TNH-2019063-2) and Osaka University (approval number: 583-2). We retrospectively reviewed medical records of patients with *Mycobacterium avium* complex pulmonary disease (MAC-PD) treated between January 2012 and June 2022. The inclusion criteria were as follows: age ≥20 years; meeting the American Thoracic Society and Infectious Diseases Society of America criteria for MAC-PD (1, 2); clarithromycin (CLR) resistance initially detected after January 2012; and availability of drug susceptibility test results for tracking and follow-up. Notably, none of the patients tested positive for human immunodeficiency virus. All patients were followed up until their last visit, death, or end of the observation period (June 30, 2022).

***Data collection***

Baseline clinical parameters, including patient age, sex, body mass index, smoking status, and comorbidities, were recorded at the time of macrolide resistance (MR) diagnosis. Additionally, data on treatment duration, antimicrobial treatment for MAC-PD, bacterial culture results, and chest computed tomography findings were obtained from medical records.

***Sputum examination***

Sputum cultures were assessed for the presence of acid-fast bacilli using either a 2% Ogawa egg medium (Japan BCG, Tokyo, Japan) or mycobacterial growth indicator tubes (Becton Dickinson, Tokyo, Japan).

***Drug susceptibility testing***

The minimal inhibitory concentrations (MICs) for CLR were measured using the broth microdilution method with the commercial BrothMIC NTM kit (Kyokuto Pharmaceutical Industrial Co. Ltd.), which complies with the Clinical and Laboratory Standards Institute (CLSI) M24-A guideline (3). MICs were determined after confirming adequate growth of the control following a 7-day incubation in a standard atmosphere at 36°C. MIC values of ≤8 µg/mL, 16 µg/mL, and ≥32 μg/mL were categorized as susceptible, intermediate, and resistant, respectively. MR was defined as CLR resistance with an MIC of ≥32 µg/mL (4-6). In patients with detected MR, the identified species and macrolide susceptibilities were routinely re-examined at least once every year after the first detection of MR and at the time of suspected recurrence or development of refractory disease in patients with positive culture isolates. Additional CLR susceptibility tests were performed using the available preserved clinical culture isolates.

***Whole genome sequencing and analyses***

Genomic DNA was extracted from MAC isolates using the DNeasy PowerSoil Pro Kit (QIAGEN, Valencia, CA, USA) following the manufacturer’s instructions. The Nextera XT DNA Library Prep Kit (Illumina, San Diego, CA, USA) was used to prepare libraries for whole genome sequence (WGS) on the MiSeq System using the 300-cycle MiSeq Reagent Kit v2 (Illumina). The mycobacterial species and subspecies were identified using the mlstverse software (7, 8). After mapping raw sequencing reads to the reference sequence using minimap2, multilocus sequence typing scores were calculated using mlstverse.Mycobacterium.db. The raw sequencing reads were mapped to 23S rRNA gene reference sequences (NC_016946.1:1639789-1642895) using minimap2 2.17. We assessed 23S rRNA gene mutations in *Escherichia coli* at positions 2058–2059, corresponding to the 23S rRNA gene reference sequence positions 2268–2269. WGS data were deposited in BioProject (PRJDB13569).

***VNTR analysis***

Mixtures were prepared from the DNA template (1 µL), 2× polymerase chain reaction (PCR) buffer (25 µL), deoxynucleoside triphosphates (10 µL), distilled water (11 µL), each primer set (both 10 mM), and polymerase (1 U, KOD-FX-Neo). In addition, primer sets for 15 *Mycobacterium* *avium* VNTR loci and 16 *M.* *intracellulare* VNTR loci were used in the VNTR analysis, as previously reported (9, 10). PCR conditions were as follows: 1 cycle of 2 min at 94°C, followed by 35 cycles of 10 s at 98°C; 30 s at 64°C; and 30 s at 68°C. The PCR products were subjected to electrophoresis on 2.5% agarose gel alongside a 100-bp DNA size ladder marker (Nacalai Tesque, Kyoto, Japan). The number of repetitions of various VNTR loci for each strain was determined and assigned using allele profile comparisons. *M. avium* complex infection of different strains was defined as present in cases when the VNTR analysis for paired *M*. *avium* or *M*. *intracellulare* strains revealed a difference at ≥1 of the 15 *M*. *avium* VNTR loci or 16 *M*. *intracellulare* VNTR loci, respectively. PCR, electrophoresis, and VNTR score calculation were performed independently by FK, YA, KH, FK, and YM. YA confirmed the consistency of the results.

***Culture experiments of paired clinical isolates***

The clinical strains stored on Ogawa medium were re-isolated using a 7H10 medium and were incubated in the medium at 37°C with shaking at 150 rpm. The isolates were picked from colonies and pre-cultivated in a 7H9 liquid medium until they reached an optical density (OD) of approximately 0.1 at 37°C with shaking at 150 rpm. Five 10-fold dilution series (n=2) were prepared from pre-cultivation using 7H9 medium in 96-well plates (3585; Corning, New York, NY, USA). The final volume in each well was 200 μL. To mimic the *in-vivo* situation, the medium containing 32 μg/mL CLR was used for culturing strains with persistent CLR-resistant phenotype, whereas the CLR-free medium was used for culturing strains with the reappearance of the CLR-susceptible phenotype. The plate was incubated for 96 h at 37°C, and the growth curve was measured using the plate reader (Multiskan GO Thermo Scientific, Waltham, MA, USA). For wells with an increase in OD, the growth rate was calculated from the growth curve using the growth curve package in R (R Software for Statistical Computing, Vienna, Austria).

***Statistical analysis***

All statistical analyses were performed using JMP Pro 17 (SAS Institute, Cary, NC, USA). Statistical significance was set at *p*-values <0.05.

**Supplemental References**

1. Daley CL, Iaccarino JM, Lange C, Cambau E, Wallace RJ, Jr., Andrejak C, Bottger EC, Brozek J, Griffith DE, Guglielmetti L, Huitt GA, Knight SL, Leitman P, Marras TK, Olivier KN, Santin M, Stout JE, Tortoli E, van Ingen J, Wagner D, Winthrop KL. 2020. Treatment of nontuberculous mycobacterial pulmonary disease: an official ATS/ERS/ESCMID/IDSA clinical practice guideline. Eur Respir J 56.

2. Griffith DE, Aksamit T, Brown-Elliott BA, Catanzaro A, Daley C, Gordin F, Holland SM, Horsburgh R, Huitt G, Iademarco MF, Iseman M, Olivier K, Ruoss S, von Reyn CF, Wallace RJ, Jr., Winthrop K, Subcommittee ATSMD, American Thoracic S, Infectious Disease Society of A. 2007. An official ATS/IDSA statement: diagnosis, treatment, and prevention of nontuberculous mycobacterial diseases. Am J Respir Crit Care Med 175:367-416.

3. Woods GL, Brown-Elliott BA, Conville PS, Desmond EP, Hall GS, Lin G, Pfyffer GE, Ridderhof JC, Siddiqi SH, Wallace RJ, Jr., Warren NG, Witebsky FG. 2011. Susceptibility Testing of Mycobacteria, Nocardiae, and Other Aerobic Actinomycetes, 2nd ed, Wayne (PA).

4. Inagaki T, Yagi T, Ichikawa K, Nakagawa T, Moriyama M, Uchiya K, Nikai T, Ogawa K. 2011. Evaluation of a rapid detection method of clarithromycin resistance genes in Mycobacterium avium complex isolates. J Antimicrob Chemother 66:722-729.

5. Morimoto K, Hasegawa N, Izumi K, Namkoong H, Uchimura K, Yoshiyama T, Hoshino Y, Kurashima A, Sokunaga J, Shibuya S, Shimojima M, Ato M, Mitarai S. 2017. A Laboratory-based Analysis of Nontuberculous Mycobacterial Lung Disease in Japan from 2012 to 2013. Ann Am Thorac Soc 14:49-56.

6. Morimoto K, Namkoong H, Hasegawa N, Nakagawa T, Morino E, Shiraishi Y, Ogawa K, Izumi K, Takasaki J, Yoshiyama T, Hoshino Y, Matsuda S, Hayashi Y, Sasaki Y, Ishii M, Kurashima A, Nishimura T, Betsuyaku T, Goto H, Nontuberculous Mycobacteriosis Japan Research C. 2016. Macrolide-Resistant Mycobacterium avium Complex Lung Disease: Analysis of 102 Consecutive Cases. Ann Am Thorac Soc 13:1904-1911.

7. Fukushima K, Matsumoto Y, Matsuki T, Saito H, Motooka D, Komukai S, Fukui E, Yamuchi J, Nitta T, Niitsu T, Abe Y, Nabeshima H, Nagahama Y, Nii T, Tsujino K, Miki K, Kitada S, Kumanogoh A, Akira S, Nakamura S, Kida H. 2023. MGIT-seq for the Identification of Nontuberculous Mycobacteria and Drug Resistance: a Prospective Study. J Clin Microbiol 61:e0162622.

8. Matsumoto Y, Kinjo T, Motooka D, Nabeya D, Jung N, Uechi K, Horii T, Iida T, Fujita J, Nakamura S. 2019. Comprehensive subspecies identification of 175 nontuberculous mycobacteria species based on 7547 genomic profiles. Emerg Microbes Infect 8:1043-1053.

9. Ichikawa K, Yagi T, Inagaki T, Moriyama M, Nakagawa T, Uchiya KI, Nikai T, Ogawa K. 2010. Molecular typing of Mycobacterium intracellulare using multilocus variable-number of tandem-repeat analysis: identification of loci and analysis of clinical isolates. Microbiology (Reading) 156:496-504.

10. Kikuchi T, Watanabe A, Gomi K, Sakakibara T, Nishimori K, Daito H, Fujimura S, Tazawa R, Inoue A, Ebina M, Tokue Y, Kaku M, Nukiwa T. 2009. Association between mycobacterial genotypes and disease progression in Mycobacterium avium pulmonary infection. Thorax 64:901-907.
